# Does Father Christmas Have a Distinctive Facial Phenotype?

**DOI:** 10.3390/vision6040071

**Published:** 2022-12-02

**Authors:** Thomas Wright, Chris Law, Ben Wright, Barry Wright

**Affiliations:** 1 Manchester Centre for Genomic Medicine, Clinical Genetics Service, Saint Mary’s Hospital, Manchester University NHS Foundation Trust, Oxford Road, Manchester M13 9WL, UK; 2 Independent Data Scientist, London N18 1QX, UK; 3 Radiotherapy Department, North Middlesex University Hospital NHS Trust, Sterling Way, London N18 1QX, UK; 4 COMIC Research, Hull York Medical School, University of York, York YO10 5NP, UK

**Keywords:** Father Christmas, Santa Claus, face, facial phenotype, facial analysis, OpenFace, dysmorphology, artificial intelligence, support vector machines

## Abstract

We investigated whether Father Christmas has a distinguishable facial phenotype by performing a cross-sectional cohort study examining the facial feature vectors of all publicly available photographs obtained from a google image search of individuals meeting our eligibility criteria presenting as Father Christmas compared with other adult and elderly bearded men. Facial feature vectors were determined using the open-source OpenFace facial recognition system and assessed by support vector machines (SVM). SVM classifiers were trained to distinguish between the facial feature vectors from our groups. Accuracy, precision, and recall results were calculated and the area under the curve (AUC) of the receiver operating characteristic (ROC) were reported for each classifier. SVM classifiers were able to distinguish the face of Father Christmas from other adult men with a high degree of accuracy and could discriminate Father Christmas from elderly bearded men but with lower accuracy. Father Christmas appears to have a distinct facial phenotype when compared to adult men and elderly bearded men. This will be reassuring to children who may be keen to recognise him but raises some interesting questions about the careful use of two-dimensional facial analysis, particularly when employed to explore the relationships between genotype and facial phenotype in a clinical dysmorphology setting.

## 1. Introduction

### 1.1. Father Christmas

Santa Claus is a renowned, festive character enriched with history and tradition [1]. Some suggest that he was based around the figure of Saint Nicholas who was born during the third century in the village of Patara, in modern-day Turkey. Father Christmas then emerged much later in the British Isles and Sinterklaas or Santa Claus in broader Western culture. Many children across the world understand this individual to be a living, generous, man who is universally recognisable [2]. Cartoon depictions of Santa Claus often present a portly, jolly, white-bearded man, wearing spectacles, dressed in a red coat and trousers detailed with a white furry collar, cuffs, dark belt and boots, carrying a bag full of gifts (and coal) for children at Christmas [1].

### 1.2. The Face of Father Christmas

A poem about this “chubby and plump” man was published by *The Sentinel* New York Newspaper in 1823 [3] and included descriptive features of his face:

*His eyes—how they twinkled! his dimples how merry!*

*His cheeks were like roses, his nose like a cherry!*

*His droll little mouth was drawn up like a bow,*

*And the beard of his chin was as white as the snow;…*

*He had a broad face and a little round belly,*

*That shook, when he laughed like a bowlful of jelly.*

Children as young as 3 in an Australian cohort were reported to self-declare their ability to authenticate the identity of Santa Claus, citing his distinctive appearance as critical [4]. What is less clear is whether the face of Santa Claus is unique or recognisable. Cardiologists have reported the face of Santa Claus to reveal itself in the para-sternal short-axis view on transthoracic echocardiography following mitral regurgitation central double-orifice surgical repair [5], suggesting this may be the case. Faces are central to our appearance, identity and identification. Understanding the face has far-reaching social, cultural, forensic and medical relevance [6]. In genomic medicine, facial characteristics are often used to establish or narrow down differential diagnoses when considering syndromic disorders [7]. The facial phenotype is highly specific for many conditions. The Human Phenotype Ontology (HPO) is a standard set of phenotypic terms organised in a hierarchical fashion that describe human disease and are used to enrich the computational analysis of genomic data [8]. Included within the HPO term vocabulary are descriptions of the face. Whilst we do not think Father Christmas has a clinical disorder, we have considered possible HPO terms related to the 1823 facial descriptions [3] (see Table 1).

### 1.3. Clinical Assessment of the Face

The clinical dysmorphology examination is informative for genomic variant interpretation [7]. Realising the challenges of dysmorphology and the value of disease-specific phenotypic data, automated computational systems have been developed to interrogate the face of patients from ordinary photographs to complement detailed clinical assessment [9]. Akin to the well-known phrase, “a picture speaks a thousand words” [10], these tools extract facial phenotypic data from photographs of the face, to generate diagnostic suggestions. This approach has been shown to be highly sensitive and specific for several conditions [6]. This is particularly relevant when considering that a typical individual undergoing whole genome sequencing usually has millions of variants compared to the reference human genome [11].

### 1.4. Assessing the Face of Father Christmas

**Figure 1:**
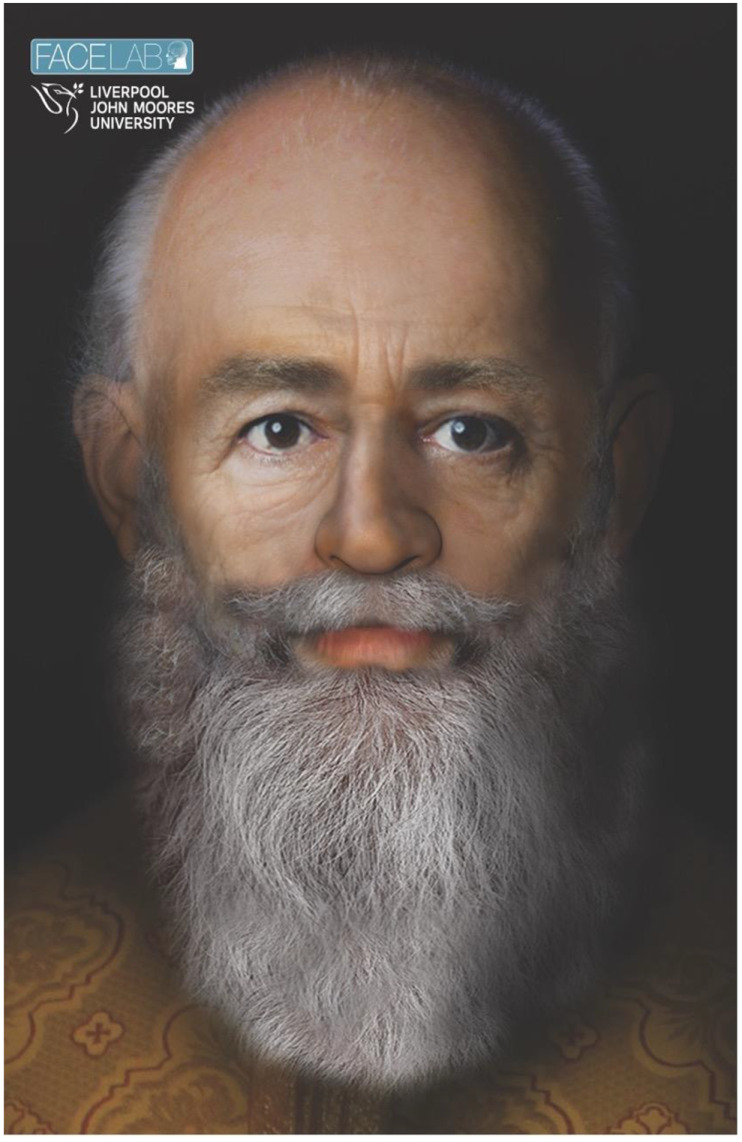
Facial depiction of Saint Nicholas following a forensic facial reconstruction approach based on skull remains *(image used with kind permission from Face Lab at Liverpool School of Art & Design, Liverpool John Moores University)*.

Previous attempts have been made to depict the face of Saint Nicholas using facial reconstruction technology from skull remains (see Figure 1). Whilst Father Christmas’ identity has been explored in the scientific literature [1,2,4,5,12], to our knowledge, the modern face of Father Christmas has never been objectively or systemically assessed. We are interested in exploring whether the modern face of Father Christmas, as presented on the internet, may be distinctive when compared to the face of other adult or elderly bearded men. We have employed face recognition assessments for this purpose.

## 2. Materials and Methods

### 2.1. Examining the Facial Phenotype of Father Christmas

We interrogated an automated algorithm that extracts facial phenotypic information from ordinary two-dimensional photographs to answer our two main questions:

1. Does Father Christmas have a facial phenotype that is distinguishable from other adult men?
2. Does Father Christmas have a facial phenotype that is distinguishable from other elderly bearded men?

Before doing this, we used photographs of Elvis Presley and Elvis Presley impersonators to validate that the facial recognition technique used in our study was able to discriminate between these distinct groups.

### 2.2. Study Group Populations

We used elements of the design employed by Roos van der Donk and colleagues [13] with additional principles from Ferry and colleagues [14], as described below. In a week in July 2022, we downloaded all eligible images following a Google Image search using a private internet browser, with search terms presented in Table 2 that comprise each of our 7 groups and corresponding search terms. Two of the authors (TW and BeW) independently applied the inclusion and exclusion criteria detailed in Table 3 to each of our groups and removed duplicates. Any discrepancies were adjudicated by a third author (BaW). Images were saved in the highest available quality in JPEG or PNG format.

### 2.3. Facial Feature Extraction

A face feature vector was determined for each image using an open-source facial recognition system, OpenFace [15]. The OpenFace pipeline was executed by CL, using a Docker container provided with the package, with further analysis performed using Google Colaboratory (Python 3.6) [16]. OpenFace runs an automated process to interpret the face(s) from an ordinary photograph by face detection, annotation of facial landmarks, and normalisation of the facial orientation through affine transformation. Following these steps, a standardised, representative, low-resolution image of the face is generated. The low-resolution facial image is then inputted into a pre-trained facial recognition deep neural network that outputs a 128-dimensional facial feature vector describing characteristics of the face(s) that are useful for facial recognition. The facial feature vector defines a position within an abstract facial feature space, where individuals with similar faces are located closer together and individuals with dissimilar faces are located further apart.

OpenFace has been demonstrated as a valid tool for numerous diverse facial recognition applications. One research group found that OpenFace was capable of detecting individuals with Koolen-de Vries syndrome (KdVS) (OMIM **#**610443), Schuurs–Hoeijmakers syndrome, (OMIM **#**615009) and PHIP-related disorder (**#**OMIM 612870) from photographs of patients’ comparative to matched controls with intellectual disability of unknown aetiology [13]. OpenFace has also been used as a tool to recognise subtle changes in facial expression present in patients with Parkinson’s disease [17].

The facial feature vectors from each of our groups were transformed using t-distributed stochastic neighbour embedding (t-SNE) to provide a visual representation of the facial feature space [18]. This approach has previously been used to present similar data related to the facial gestalt of positive and negative controls in the field of Genomic Medicine [13].

### 2.4. Support Vector Machine

To test our hypothesis that Father Christmas has a distinct facial phenotype, we trained support vector machine (SVM) classifiers to distinguish the study groups in the facial feature space [19]. SVM classifiers were chosen as they have been shown to outperform traditional classification and distance measurement methods in facial recognition statistical assessments [20]. Our groups were randomly split into training and testing sets with a test size of 30%. Training and hyperparameter tuning were then performed on the training set through grid search cross-validation. Accuracy, precision, and recall results were calculated using the test set, and the area under curve (AUC) of the receiver operating characteristic (ROC) and corresponding *p* values [21] were reported.

We first validated this approach using Elvis Presley and Elvis Presley Impersonator groups. SVM classifiers were implemented to distinguish between points in the facial feature space labelled (1) Elvis Presley and Adult Man, (2) Elvis Presley Impersonator and Adult Man, and (3) Elvis Presley and Elvis Presley Impersonator. The same approach was used to train classifiers to distinguish our Total Father Christmas group from (1) Adult Man and (2) Elderly Bearded Man. An additional SVM classifier was trained to distinguish between Father Christmas and Santa Claus to explore the unlikely possibility that they may occupy different facial feature spaces. We then applied each of the SVM classifiers to the facial feature vector of Face Lab’s facial depiction of Saint Nicholas to determine which group Saint Nicholas is predicted to belong to.

### 2.5. Facial Averages

We constructed a representative facial image for each group. Facial landmark annotation and alignment were performed using the OpenFace package. All images were averaged to generate a facial average for each group.

## 3. Results

### 3.1. Groups

**Figure 2:**
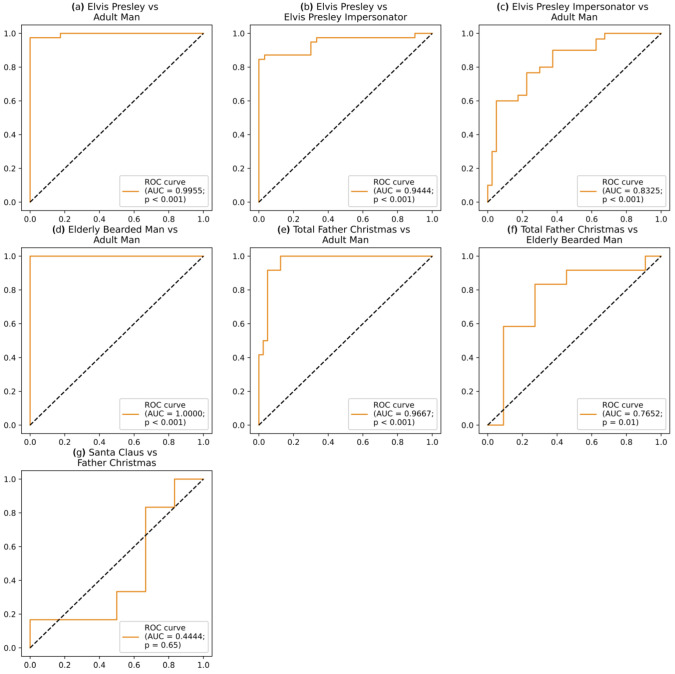
Receiver operating characteristic (ROC) curves for the support vector machine (SVM) classifiers trained to distinguish our groups: (**a**) Elvis Presley and Adult Man, (**b**) Elvis Presley and Elvis Presley Impersonator, (**c**) Elvis Presley Impersonator and Adult Man, (**d**) Elderly Bearded Man and Adult Man, (**e**) Total Father Christmas and Adult Man, (**f**) Total Father Christmas and Elderly Bearded Man, and (**g**) Santa Claus and Father Christmas. Areas under these ROC curves (AUC) are shown in the legends.

Table 4 shows the number of images where facial feature vectors were generated for each group following google image searches. Father Christmas and Santa Claus were treated as one group (Total Father Christmas) after we found that the receiver operating characteristic curve closely tracked the bisecting line showing that they were from the same group (see Figure 2g). This was confirmed by our SVM classifier which was unable to distinguish any difference between Father Christmas and Santa Claus.

### 3.2. Facial Feature Space

**Figure 3:**
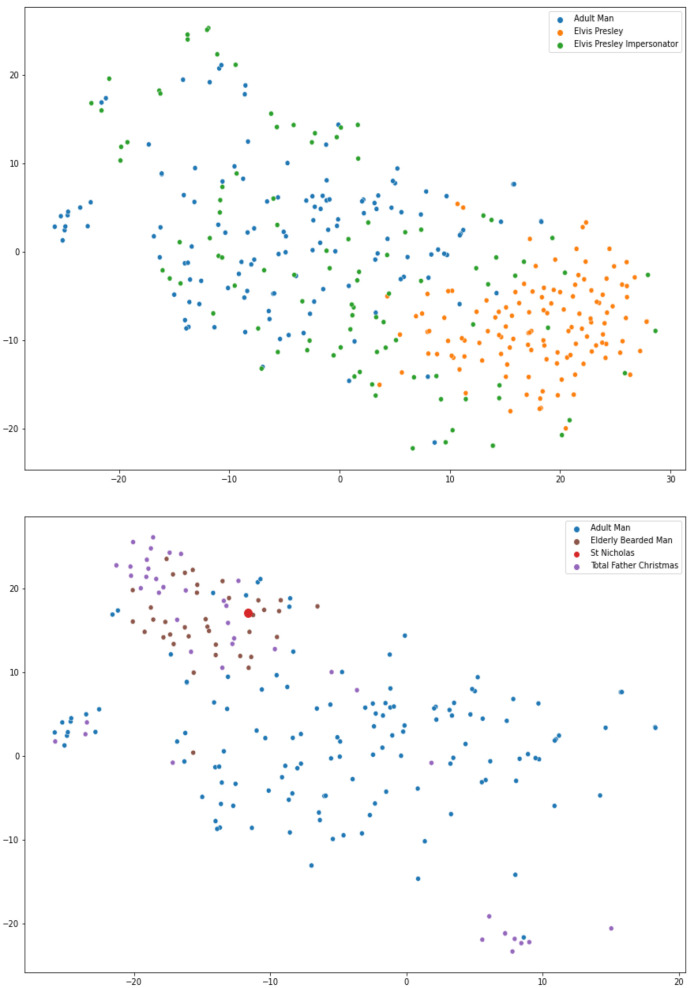
Visual representation* of the distribution of the facial feature vectors. Upper Panel: Adult Man (blue circles), Elvis Presley (orange circles), and Elvis Presley Impersonator (green circles) groups. Lower Panel: Adult Man (blue circles), Elderly Bearded Man (brown circles), and Total Father Christmas (purple circles) groups. The facial feature vector of Face Lab’s Saint Nicholas is also presented (larger red circle). *The full 128-dimensional feature vectors generated by OpenFace are presented visually in this figure by reducing to 2-dimensional vectors using t-distributed stochastic neighbour embedding (t-SNE), as described in the methods section.

Figure 3 show a visual representation of the facial feature space for the comparisons as described in the methods section.

### 3.3. Support Vector Machine Results

**Figure 4:**
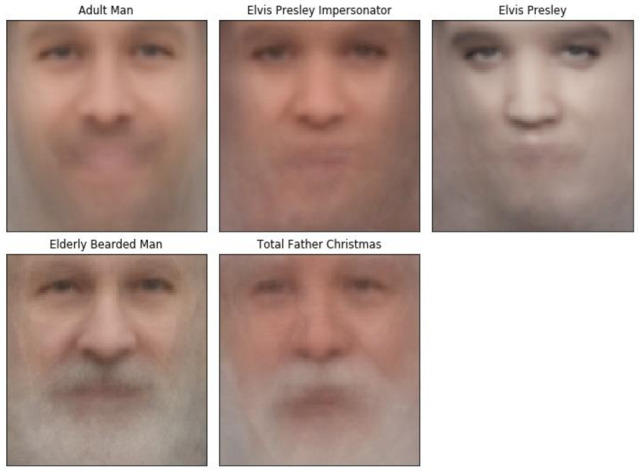
Facial Averages for each group: Adult Man (upper left panel), Elvis Presley Impersonator (upper middle panel), Elvis Presley (upper right panel), Elderly Bearded Man (lower left panel) and Total Father Christmas (lower middle panel).

SVM Classifier results are presented in Table 5. ROC curves were plotted for each comparison (see Figure 2). We applied the classifier trained to distinguish Total Father Christmas from Adult Man to the facial feature vector of Face Lab’s depiction of Saint Nicholas. The SVM classifier predicted that Saint Nicholas’ face belongs to the Adult Man class rather than the Total Father Christmas class. We then applied the SVM classifier trained to distinguish Elderly Bearded Man from Adult Man and found that Saint Nicholas’ face was predicted to belong to the Elderly Bearded Man class. Finally, we generated facial averages in the OpenFace package for each of our groups, as shown in Figure 4.

## 4. Discussion

### 4.1. Does Father Christmas have a Distinctive Facial Phenotype?

Clear differences were found between our groups containing Total Father Christmas and Adult Man using facial feature vector distributions and ROC curve analysis suggesting a clear facial phenotype for our Total Father Christmas group. This is also the case for the Total Father Christmas and Elderly Bearded Man groups although performance was lower in this comparison. This may be because Father Christmas facial phenotype clusters mainly as a subgroup within elderly bearded men (see t-SNE distribution in Figure 3). Other explanations such as the presence of facial hair impeding facial feature discrimination are possible [22] or we may be observing an artefactual or a chance finding.

### 4.2. Validation of the Methods Used

We sought to separately test the reliability of the face recognition methodology used in our study and were able to demonstrate statistically significant differences between the Elderly Bearded Man and Adult Man groups. The facial features of our groups containing Elvis Presley, Elvis Presley impersonators and Adult Man were also distinctly different. This confirms that the OpenFace facial recognition approach employed here can carry out high-level facial feature discrimination. The SVM classifier validation experiment (see Table 5) was able to discriminate between what one would expect to be a genetically diverse group (Elvis Presley impersonators) who have a distinct facial phenotype from adult men, which is perhaps demonstrating the presence of selection bias in the use of datasets.

### 4.3. The Origins of Father Christmas

We were intrigued to find that the SVM classifier predicted that Saint Nicholas’ face belongs to the Elderly Bearded Man group, rather than the Father Christmas group, suggesting a less prominent association than proposed by other authors [1]. A recent paper has made a bold claim, that rejects Santa Claus and/or Father Christmas as tracing back to Saint Nicholas and instead proposes that he originated from a more recent man called Walter Clement Shields, who organised reindeer fairs in Alaska in the early twentieth century and delivered gifts on a reindeer sleigh [23]. We were unable to support this theory in our study following SVM classification, which predicted Mr Shield’s face belongs to the Adult Man group [24]. Perhaps a more likely explanation is that rich cultural and increasingly commercial traditions in Europe, North America and across the globe (long after Saint Nicholas’ birth in the third century) have iteratively promulgated ideas about, and increasingly uniform images of Father Christmas and Santa Claus.

### 4.4. Consideration of Bias

The issue of bias is worthy of further consideration given our finding that the facial features of Father Christmas appear to be distinct from elderly bearded men. This finding could be confirming that Father Christmas is real, but other possibilities should be considered. Could selection bias related to facial characteristics be operating [25]? This may be a result of self-selection or selective invitation to the role by appearance. It is also possible that facial disguise, distortion, or manipulation such as the use of make-up, might be playing a role [26]. Our results also raise some important questions about the role and use of facial recognition software and inherent or unknown bias that result from algorithm establishment, algorithm training or database usage [27]. This includes a bias related to the database or training set being used by the system [9]. Our study relies upon already identified pictures of Father Christmas. A parallel use of facial analysis takes place in Genomic Medicine to complement the clinical dysmorphology assessment. Automated facial analysis of patient two-dimensional photographs can be used as tools to explore facial attributes in relation to genetic conditions [9,28]. These systems can have around 90% sensitivity and specificity when exploring facial phenotypes [29], although they are also vulnerable to various biases including those related to selection, gender and ethnicity [27,30]. Clinicians may omit faces (e.g., outliers or typical faces) that do not match testing paradigms or their training experience (e.g., conforming to their beliefs about typical facial features in a particular condition). In this way, results could be influenced by factors such as training, experience and possible unconscious or other bias.

### 4.5. Other Limitations

Limitations of our study include the reliance on a particular set of available online images to establish each of our groups that may have various factors influencing their presence there. In addition, we were only able to find relatively small numbers of eligible photographs for our groups, due to our relatively strict exclusion criteria and the recognised challenge of facial feature vector extraction from low-resolution and complex facial images. Another limitation of our study is the use of a single facial recognition assessment algorithm, OpenFace, to assess the facial feature vectors of our dataset. Whilst examining our photographic images with an alternative, additional deep learning-based face recognition system may be considered as optimal, it was beyond the scope of this study to provide this comparison. This could be evaluated in future studies. Reassuringly, OpenFace has been extensively validated [13,15,17] and has been shown to be comparable to alternative approaches [31]. A further study found that OpenFace was the best-performing open-source facial recognition algorithm and has been shown to be useful with small sample sizes, similar to those used in our study [32].

It is difficult to avoid some aspects of human selection for facial analysis systems, which are required to develop algorithms and computerised neural networks. Our study relied on available prevalent images which shone a light on concerns raised in the face recognition literature about biases that may relate to race, ethnicity and skin colour [27,30]. Data curation issues such as maintaining the quality of the data for the intended purpose are important. The issues related to bias in this evolving field are well recognised, and researchers are busy exploring ways to minimise these [9,27,30]. Attempts have been made to blind algorithms to known bias where possible [30], improving methods to measure bias and develop an awareness of it [27], curating deep convolutional neural networks that avoid the need to include positive test cases [9], which may have inherent biases in training sets [9], and increasing computational power by using approaches that generate larger numbers of comparative data parameters, such as in 3D modelling [33]. This field is making large strides towards improved facial assessment methodologies. We recommend caution in the over-reliance on these facial assessment technologies for clinical use, which are vulnerable to a range of different sources of bias. This is particularly important if the results of automated facial assessments are intended to be used to provide phenotypic evidence to support a diagnosis in the context of genomic variant interpretation [7,9].

### 4.6. Study Implications and Applications

Our study demonstrates a novel use of face recognition, by examining the distinctiveness of the face of individuals presented on google images as Father Christmas. It is unclear whether our finding that Father Christmas has a distinctive face may be replicated in other cohorts of Father Christmas. Further research is needed to examine this, such as the Father Christmas cohort attending the World Santa Claus Congress. This is an annual event in Denmark established in 1963 and is usually attended by up to 500 individuals professionally employed as Father Christmas from around the world [2]. In addition, it would be interesting to examine the inter- and intra-facial variability of related festive characters, such as Papa Noël, Tomte, Julenissen, Ded Moroz, Sinterklaas and Los Reyes Magos. Further research is required to explore whether our finding that Father Christmas has a distinct face may extend to other folklore characters, such as The Tooth Fairy, Robin Hood or Tom Thumb [34].

In our study, we validated our approach by comparing the face of Elvis Presley with Elvis Presley impersonators. This is an additional novel use of facial recognition. Similar assessments could be applied on a large scale to determine and measure the objective facial vector similarity between an impersonator comparative to the individual being impersonated. There is potential to provide outputs such as a “lookalike score” based on facial feature vector similarities which may be attractive to the impersonator and the consumer in this large and culturally important industry, where measures of authenticity are highly valued [2,35].

## 5. Conclusions

This study suggests that Father Christmas has a distinctive face that can be discriminated from other adult men and elderly bearded men, lending weight to the widely held belief amongst children that he is a real person and recognisable anywhere [36].

## Figures and Tables

**Table 1:** Facial descriptors of Father Christmas from 1823 and possible corresponding HPO terms.

1823 Facial Descriptor	HPO Term	Term ID
Twinkled eyes	Epiphora	HP:0009926
Merry dimples	Skin dimple Chin dimples	HP:0010751 HP:0010781
Cheeks like roses	Telangiectases of the cheeks Facial erythema Malar rash	HP:0007421 HP:0001041 HP:0025300
Nose like a cherry	Bulbous nose	HP:0000414
Droll little, bow-like mouth	Narrow mouth Exaggerated cupid’s bow	HP:0000160 HP:0002263
Beard as white as snow	Facial hypertrichosis Hypopigmented hair	HP:0002219 HP:0011364
Broad face	Broad face	HP:0000283

**Table 2:** Study group populations and corresponding google image search terms.

Groups		Search Terms
Validation groups	Elvis Presley	“Elvis Presley”
	Elvis Presley impersonator	“Elvis Presley impersonator”
Test groups	Father Christmas	“Father Christmas”
	Santa Claus	“Santa Claus” “Santa”
	Total Father Christmas	“Father Christmas” “Santa Claus” “Santa”
Control groups	Adult man	“Adult man”
	Elderly bearded man	“Elderly bearded man”

**Table 3:** Facial image inclusion and exclusion criteria for study groups.

Inclusion Criteria	Exclusion Criteria
Photographic images	Cartoons and other non-photographic images
Frontal view of face	
Labelled as the individual specified by the search term	
Containing one person	Group photographs
Bearded and non-bearded individuals	Face obscured by physical or digital covering(s) (e.g., sunglasses, face mask, digital watermark)
Both eyes open	
	Face not detectable by OpenFace algorithm

**Table 4:** Groups sizes following application of inclusion and exclusion criteria and generation of facial feature vectors by OpenFace.

Group	N
Adult Man	132
Elderly Bearded Man	37
Elvis Presley Impersonator	100
Elvis Presley	128
Father Christmas	21
Santa Claus	22
Total Father Christmas	43

**Table 5:** Accuracy, precision, recall and AUC for SVM classifiers for study group comparisons.

Comparison Groups	Accuracy	Precision	Recall	AUC (*p* Value)
Elvis Presley vs. Adult Man	0.9620	1.0000	0.9231	0.9955 (<0.001)
Elvis Presley vs. Elvis Presley Impersonator	0.8116	0.8095	0.8718	0.9444 (<0.001)
Elvis Presley Impersonator vs. Adult Man	0.7857	0.8571	0.6000	0.8325 (<0.001)
Elderly Bearded Man vs. Adult Man	0.9804	0.9167	1.0000	1.0000 (<0.001)
Total Father Christmas vs. Adult Man	0.9231	0.8333	0.8333	0.9667 (<0.001)
Total Father Christmas vs. Elderly Bearded Man	0.7391	0.7500	0.7500	0.7652 (0.01)
Santa Claus vs. Father Christmas	0.5833	0.5556	0.8333	0.4444 (0.64)

## Data Availability

Full reproducible code is available at: https://github.com/claw89/fc_phenotype (accessed on 21 November 2022). Requests for access to the photograph images from each study group should be made to the corresponding author.
